# A systematic review and meta-analysis of moxibustion for chronic prostatitis

**DOI:** 10.1097/MD.0000000000036742

**Published:** 2023-12-15

**Authors:** Xi-wen Yu, Cheng-si Wang, Xiao-hong Yu

**Affiliations:** a Department of Acupuncture and Moxibustion, Baicheng Medical College, Baicheng, China; b College of Mathematical Sciences, Shanghai Jiaotong University, Shanghai, China; c Second Ward of Cardiology Department, First Affiliated Hospital of Heilongjiang University of Chinese Medicine, Harbin, China.

**Keywords:** chronic prostatitis, meta-analysis, moxibustion, systematic review

## Abstract

**Background:**

Chronic prostatitis (CP) is a common condition that affects many individuals. Previous clinical trials have explored the use of moxibustion as a potential treatment for CP. However, the evidence on the effectiveness of moxibustion for CP remains limited. Therefore, this study aimed to comprehensively assess the effects of moxibustion for CP.

**Methods:**

In order to gather relevant and up-to-date information, we conducted a systematic literature search of databases including Cochrane Library, PUBMED, EMBASE, CNKI, and Wangfang from inception until June 30, 2023. Only randomized clinical trials (RCTs) that investigated the use of moxibustion for CP were included in this study. The primary outcomes of interest were the National Institutes of Health-Chronic Prostatitis Symptom Index (NIH-CPSI) scores and the overall response rate. To evaluate the quality of the included studies, we used the Cochrane risk-of-bias tool.

**Results:**

After analyzing the data from 8 RCTs involving a total of 664 patients, we found significant differences in NIH-CPSI scores between moxibustion and other treatment modalities. Specifically, when compared with herbal medicine, moxibustion was associated with a mean difference (MD) of −1.78 in NIH-CPSI scores (95% confidence interval [CI] [−2.78, −0.78], *P* < .001), and when compared with western medicine, moxibustion was associated with a MD of −5.24 in NIH-CPSI scores (95% CI [−7.80, −2.67], *P* < .08). In terms of the overall response rate, moxibustion was found to be superior to herbal medicine, with a MD of 2.36 (95% [19, 4.67], *P* = .01). Additionally, when moxibustion was combined with herbal medicine, it yielded a higher overall response rate with a MD of 4.07 (95% CI [1.54, 10.74], *P* = .005) compared to herbal medicine alone. Moxibustion also outperformed western medicine in terms of the overall response rate, with a MD of 4.56 (95% CI [2.24, 9.26], *P* < .001).

**Conclusion:**

Based on the findings of this study, moxibustion appears to be a potentially efficacious treatment for CP. The results suggest that moxibustion can improve NIH-CPSI scores and overall response rate in patients with CP. However, further high-quality studies are needed to validate these results and establish the long-term effects of moxibustion as a treatment for CP.

## 1. Introduction

Chronic prostatitis (CP) is a frequently observed condition in males, marked by the presence of pain and urinary symptoms. These symptoms can vary in intensity and duration and may include discomfort or pain in the pelvis, perineum, lower back, or genitals, as well as urinary urgency, frequency, or hesitancy.^[1–3]^ Studies conducted on a large scale have demonstrated that the prevalence of CP falls between 4.5% and 9%, indicating that a significant portion of the male population is affected by this disorder.^[4–6]^ Moreover, research has also indicated that as men age, the likelihood of experiencing recurrence of CP increases drastically, with recurrence rates soaring up to 50%.^[4–6]^ Despite the relatively high prevalence of CP, managing this condition in clinical practice poses significant challenges for healthcare professionals. The complex nature of CP makes it difficult to diagnose accurately and treat effectively. Furthermore, the wide range of symptoms and their varying severity from patient to patient necessitate an individualized approach to management. The lack of standardized diagnostic criteria and the limited understanding of the underlying mechanisms contributing to the development and persistence of CP add further complexity to its management. To address the management challenges associated with CP, healthcare providers must adopt a multidisciplinary approach that involves collaboration between urologists, pain specialists, physiotherapists, and psychologists. This integrated approach allows for a comprehensive evaluation of patients, taking into account their physical, psychological, and social factors. By addressing the diverse aspects of CP, healthcare professionals can develop personalized treatment plans that may include a combination of pharmacological interventions, physical therapy, psychological counseling, and lifestyle modifications. Moreover, raising awareness about CP among both healthcare providers and the general public is crucial for early detection and timely intervention. This can be achieved through educational programs, public health campaigns, and support groups. By improving knowledge and understanding of CP, individuals affected by this condition can seek appropriate medical care and receive the necessary support to better manage their symptoms and improve their quality of life. In conclusion, CP poses significant challenges in clinical practice due to its high prevalence, recurrence rates, and the complexity of its symptoms. Addressing these challenges requires a multidisciplinary approach, personalized treatment plans, and increased awareness. With these strategies in place, healthcare professionals can better support individuals with CP and enhance their overall well-being.

Currently, the available treatments for CP primarily consist of alpha-blockers, anti-inflammatory drugs, physical therapy, anxiolytics, and antidepressants.^[7]^ While these treatment options exist, they often fail to provide satisfactory results for patients, leaving their symptoms unresolved. Additionally, these conventional treatments pose considerable risks of adverse events, which further complicate the well-being of patients. Consequently, there is an urgent and critical requirement for the development and implementation of alternative therapies that are both safe and more efficacious in managing the symptoms and overall condition of CP patients.

Complementary and alternative therapies, such as acupuncture and moxibustion, have gained significant popularity in the treatment of pain, pressure ulcer, and urinary disorders, including conditions such as urinary incontinence, urinary retention, and CP.^[8–15]^ Among these therapies, moxibustion has emerged as a particularly important management option. Studies have reported that moxibustion has been found to have positive effects on the blood circulation of the prostate.^[16,17]^ By stimulating the acupoints and applying heat, moxibustion can effectively increase blood flow to the prostate gland, thereby improving its function and relieving the spasm of smooth muscles in the area. This enhanced blood circulation contributes to the alleviation of both local symptoms, such as urinary difficulties and pain, as well as systemic functions associated with prostate health.^[16]^ Furthermore, moxibustion has been shown to have anti-inflammatory properties.^[17]^ It can effectively reduce the secretion of inflammatory substances in the body, thereby decreasing the levels of inflammation in the prostate. By promoting the absorption of inflammatory exudates, moxibustion helps to eliminate inflammation and speed up the healing process. This anti-inflammatory effect is crucial in managing conditions such as prostatitis, where inflammation plays a significant role in exacerbating symptoms. Overall, moxibustion ability to improve blood circulation, relieve smooth muscle spasms, and reduce inflammation makes it a valuable therapeutic modality in improving prostate health.

Previous studies have delved into the potential benefits of moxibustion for patients suffering from CP.^[18–25]^ These studies have explored various aspects of moxibustion treatment, including its impact on pain reduction, improvement in urinary symptoms, and overall quality of life for individuals with prostatitis. However, despite these individual studies, no comprehensive systematic review or meta-analysis has been conducted to specifically evaluate the effects of moxibustion on prostatitis. Although a previous similar study has addressed the moxibustion for CP, it is a protocol without results.^[26]^ Therefore, the main objective of this study is to address this research gap and provide a comprehensive assessment of the specific effects of moxibustion for the treatment of prostatitis. By analyzing and synthesizing the existing literature, this study aims to shed light on the potential benefits of moxibustion as a therapeutic intervention for prostatitis patients. The findings of this study will not only contribute to the existing body of knowledge but also provide valuable insights for healthcare professionals and patients seeking alternative treatment options for managing prostatitis.

## 2. Methods

### 2.1. Ethical approval statement

This systematic review analyzed secondary data from published studies. Thus, no ethical approval is needed in this study.

### 2.2. Eligibility criteria

The inclusion criteria for this study were as follows: The patients included in the study had to be over 18 years old and diagnosed with prostatitis. Only randomized controlled trials (RCTs) were considered for inclusion. In the treatment group, patients received either moxibustion alone, moxibustion combined with herbal medicine, or moxibustion combined with western medicine. In the control group, patients received the same herbal medicine or western medicine as the treatment group. There were no restrictions on race or sex for the patients included in the study.

The exclusion criteria for this study were as follows: Studies that were not relevant to the topic, including non-clinical studies, uncontrolled trials, non-RCTs and quasi-RCTs, were excluded. Studies that did not focus on moxibustion, combined therapy, or made incorrect comparisons were excluded. Additionally, duplicated studies that had already been included were also excluded. Studies that had incomplete information, such as missing data or incomplete reporting, were also excluded.

### 2.3. Search strategy for eligible records

The search strategy for eligible records involved searching through various databases including the Cochrane Library, PUBMED, EMBASE, CNKI, and Wangfang. The search was conducted from the inception of these databases to June 30, 2023. Only RCTs on moxibustion for CP were included in the study. The search terms used included a combination of keywords such as prostatitis, prostatodynia, prostatalgia, prostate syndrome, chronic pelvic pain syndrome, chronic pelvic floor pain, moxibustion, and mugwort. Additionally, other sources of relevant records, such as the reference lists of associated reviews, were also identified and considered.

### 2.4. Outcome measurement

The outcomes assessed in this study were the National Institutes of Health-Chronic Prostatitis Symptom Index (NIH-CPSI)^[27]^ and overall response rate. The NIH-CPSI is a comprehensive survey specifically designed to assess and evaluate various symptoms associated with CP.^[27]^ This survey covers 9 different domains, each representing a specific aspect of the condition symptoms. The domains in the NIH-CPSI include pain or discomfort in the perineum, urethra, or genitals; urinary symptoms such as frequency, urgency, and weak stream; pain or discomfort during or after ejaculation; and impact of the condition on quality of life. To calculate the overall score on the index, each domain is assigned a score ranging from 0 to 5, with 0 indicating no symptoms and 5 representing the most severe symptoms. By summing up the scores from all 9 domains, the total score can range from 0 to 43. A higher total score on the NIH-CPSI signifies more severe clinical symptoms and indicates a greater impact of CP on the individual daily life. This index serves as a valuable tool in assessing the severity of symptoms and tracking the progress of treatment interventions for CP.

Overall response rate was defined as calculated by adding the number of patients who have achieved complete recovery, significant improvement, and general improvement, and then dividing it by the total number of patients. This result is then multiplied by 100% to express it as a percentage.

### 2.5. Study selection, data extraction, and risk of bias assessment

Two authors independently conducted the selection of studies, extraction of data, and assessment of risk of bias. Any disagreements were resolved through discussion with a third author. The following data were collected for each study: title, first author, publication date, patient age, gender, sample size, details of randomization, blinding, allocation, management dosage, frequency, and outcomes. The Cochrane risk-of-bias tool was used to evaluate the risk of bias in each included trial.^[28]^

### 2.6. Statistical analysis

Statistical analysis was performed using RevMan 5.4 software. Continuous data were analyzed as mean differences (MD) with 95% confidence intervals (CI), while dichotomous data were presented as odds ratios with 95% CI. The *I*² index was used to assess statistical heterogeneity across the eligible RCTs. Depending on the degree of heterogeneity, a random-effects or fixed-effects model was applied to combine the outcome data.

## 3. Results

### 3.1. Study selection

**Figure 1.**
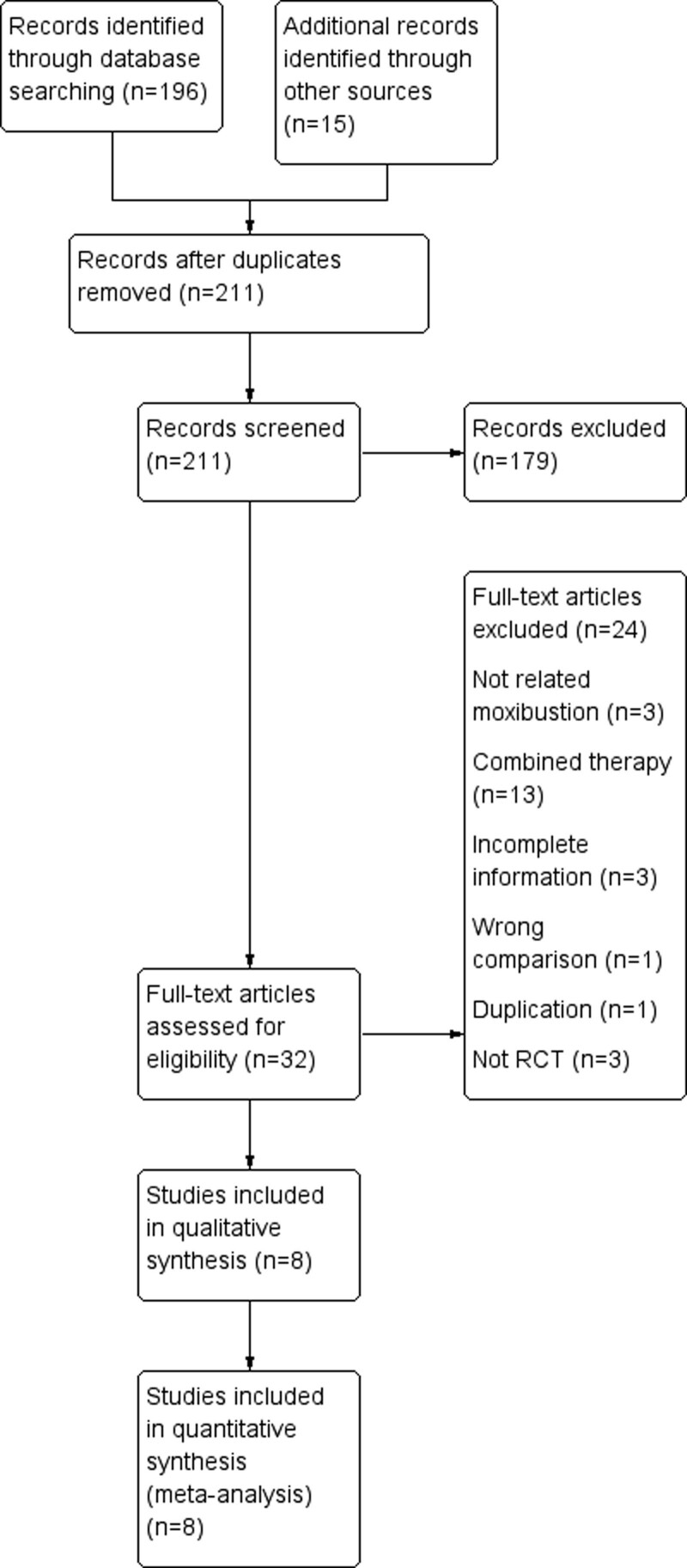
Flow diagram of study selection.

A total of 211 records were identified during the study (Fig. 1). Out of these, 179 studies were found to be irrelevant and were excluded. The remaining 32 full-text studies were carefully read and assessed for eligibility. Among them, 24 articles were excluded for various reasons such as not being related to moxibustion or combined therapy, incomplete information, incorrect comparisons, duplication, and not being RCTs. Finally, 8 trials involving 664 patients were included for analysis (Fig. 1).

### 3.2. Study characteristics

Table 1 summarizes the general characteristics of all the included RCTs. All studies were conducted in China. No data of the sample size of the trials ranged from 60 to 120. The age of the participants in the treatment group varied from (25.6 ± 8.27) to (45.12 ± 7.65) years, while in the control group it ranged from (26.3 ± 7.57) to (45.32 ± 8.15) years. The follow-up duration in the studies was either 4 or 6 weeks. As for herbal medicine, it included Qianliexin capsule, Qianlie Red-deer formula, Qianliekang, and Sancaoan decoction. As for western medicine, it consisted of alpha-blocker amitriptyline, levofloxacin hydrochloride, tamsulosin hydrochloride capsules, mirabiline, and senitong tablets. The outcomes measured in the trials included the NIH-CPSI and overall response rate. In addition, we were unable to determine the cause of CP in this study because we were unable to locate any relevant data from the original trial.

**Table 1:** General characteristics of included studies.

Study	Country	No. of patients (T/C)	Age (yr, T/C)	Intervention	Control	Outcomes	Follow-up
Chen 2008	China	48/40	T:36.78 ± 6.41; C:35.84 ± 7.23	Moxibustion	Western medicine	NIH-CPSI; overall response rate	4 wk
Kang 2015	China	60/60	T:34.23 ± 3.28; C:35.26 ± 4.17	Moxibustion	Herbal medicine	NIH-CPSI; overall response rate	4 wk
Ma 2012	China	30/30	T:30.6 ± 10.7; C:31.2 ± 10.6	Moxibustion	Western medicine	NIH-CPSI; overall response rate	4 wk
Mi 2019	China	50/50	T:36.44 ± 8.29; C:34.08 ± 7.04	Moxibustion + herbal medicine	Herbal medicine	NIH-CPSI; overall response rate	6 wk
Qiu 1999	China	30/30	T:38.40 ± 7.77; C:38.20 ± 7.74	Moxibustion	Herbal medicine	Overall response rate	4 wk
Wang 2007	China	30/30	T:25.6 ± 8.27; C:26.3 ± 7.57	Moxibustion	Western medicine	NIH-CPSI; overall response rate	4 wk
Wang 2008	China	58/30	T:26.1 ± 7.87; C:26.4 ± 8.36	Moxibustion	Western medicine	NIH-CPSI; overall response rate	4 wk
Xie 2022	China	30/30	T:45.12 ± 7.65; C:45.32 ± 8.15	Moxibustion + herbal medicine	Herbal medicine	NIH-CPSI; overall response rate	4 wk

C = control group, NIH-CPSI = National Institutes of Health-Chronic Prostatitis Symptom Index, T = treatment group.

### 3.3. Risk of bias assessment

**Figure 2.**
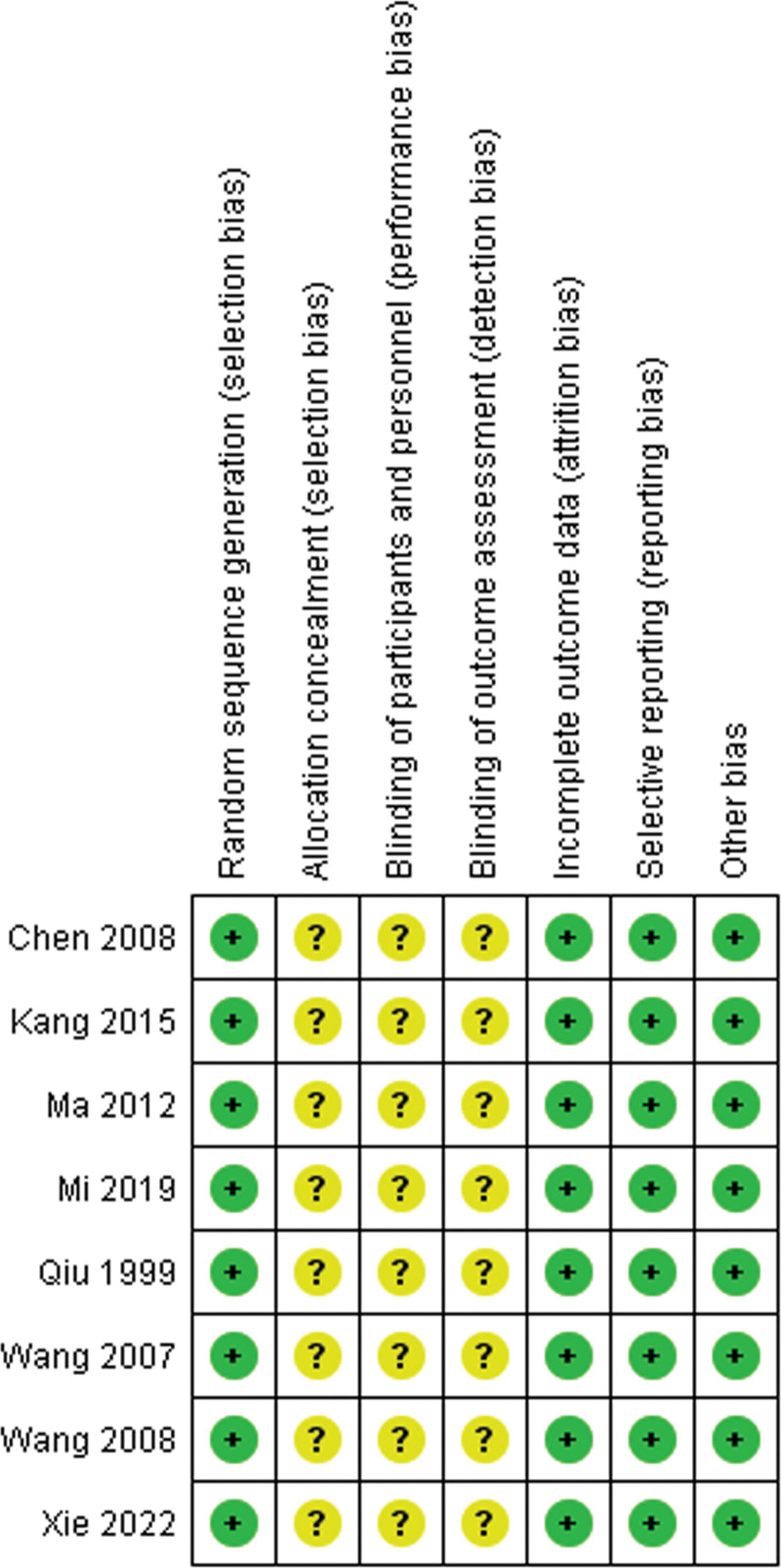
Risk of bias summary.

Figure 2 displays the risk of bias assessment for all the included RCTs. All 8 trials provided adequate details regarding random sequence generation.^[12–19]^ Additionally, all studies sufficiently reported on incomplete outcome data, selective reporting, and other forms of bias. However, none of the studies clearly reported on allocation concealment or blinding of participants, personnel, and outcome assessment^[12–19]^ (Fig. 2).

### 3.4. Moxibustion versus herbal medicine

#### 3.4.1. Meta-analysis of NIH-CPSI.

**Figure 3.**
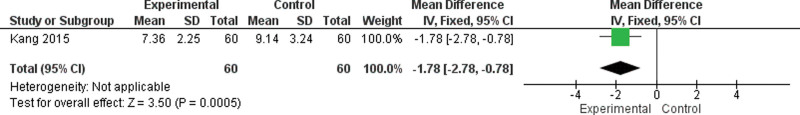
Moxibustion versus herbal medicine/meta-analysis of NIH-CPSI. NIH-CPSI = National Institutes of Health-Chronic Prostatitis Symptom Index.

One study involving 120 patients assessed the NIH-CPSI. The results showed a statistically significant difference in the NIH-CPSI between the 2 groups (MD = −1.78, 95% CI [−2.78, −0.78], *P* < .001; Fig. 3).^[13]^

#### 3.4.2. Meta-analysis of overall response rate.

**Figure 4.**
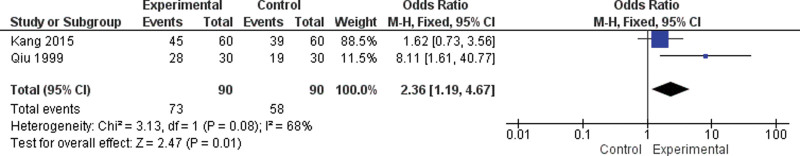
Moxibustion versus herbal medicine/meta-analysis of overall response rate.

Two RCTs involving 180 patients evaluated the overall response rate. The results indicated a statistically significant difference in the overall response rate between the 2 groups (MD = 2.36, 95% CI [1.19, 4.67], I^2^ = 68%, *P* = .01; Fig. 4).^[13,16]^

### 3.5. Moxibustion plus herbal medicine versus herbal medicine

#### 3.5.1. Meta-analysis of NIH-CPSI.

**Figure 5.**
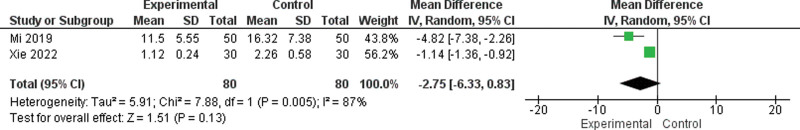
Moxibustion plus herbal medicine versus herbal medicine/meta-analysis of NIH-CPSI. NIH-CPSI = National Institutes of Health-Chronic Prostatitis Symptom Index.

Two trials with 160 patients investigated the NIH-CPSI (Fig. 5). The results showed no statistically significant difference in the NIH-CPSI between the 2 groups (MD = −2.75, 95% CI [−6.33, 0.83], I^2^ = 87%, *P* = .13; Fig. 5).^[15,19]^

#### 3.5.2. Meta-analysis of overall response rate.

**Figure 6.**
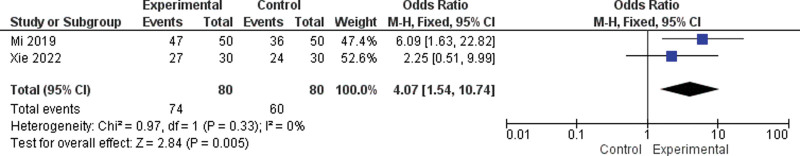
Moxibustion plus herbal medicine versus herbal medicine/meta-analysis of overall response rate.

Two RCTs with 180 subjects assessed the overall response rate. The results indicated statistically significant differences between the 2 groups (MD = 4.07, 95% CI [1.54, 10.74], I^2^ = 0%, *P* = .005; Fig. 6).^[15,19]^

### 3.6. Moxibustion versus western medicine

#### 3.6.1. Meta-analysis of NIH-CPSI.

**Figure 7.**
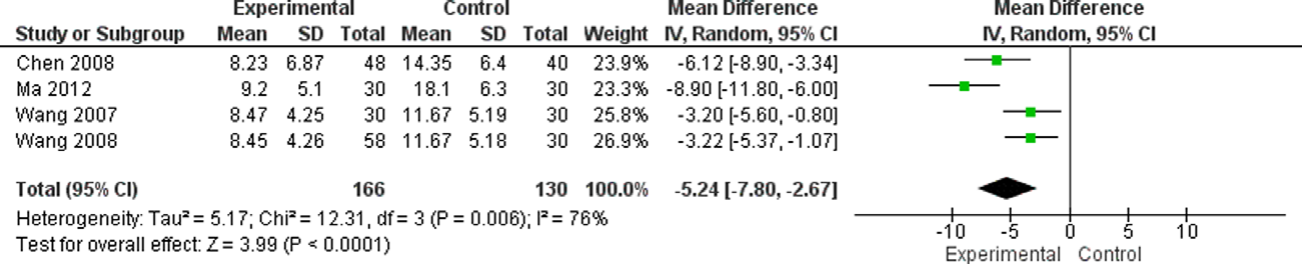
Moxibustion versus western medicine/meta-analysis of NIH-CPSI. NIH-CPSI = National Institutes of Health-Chronic Prostatitis Symptom Index.

Four trials with 296 patients investigated the NIH-CPSI (Fig. 7).^[12,14,17,18]^ The results showed a significant difference in the NIH-CPSI between the moxibustion and western medicine groups (MD = −5.24, 95% CI [−7.80, −2.67], *P* < .08; Fig. 7).

#### 3.6.2. Overall response rate.

**Figure 8.**
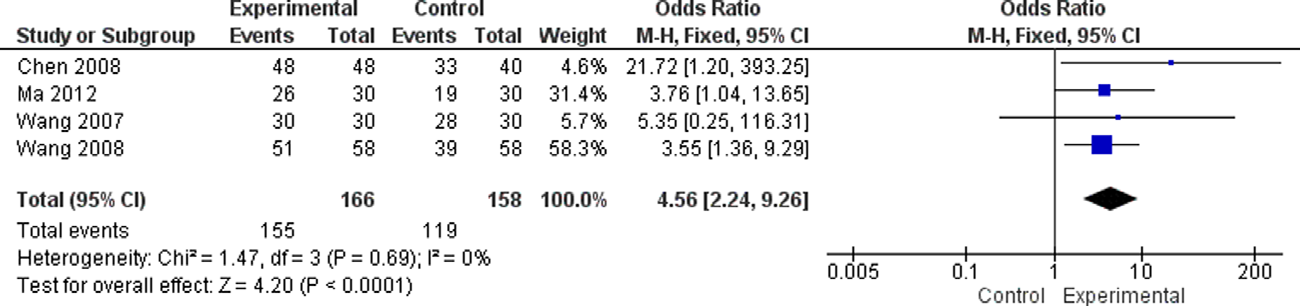
Moxibustion versus western medicine/meta-analysis of overall response rate.

Four RCTs involving 296 subjects assessed the overall response rate (Fig. 8).^[12,14,17,18]^ The results demonstrated a significant difference in the overall response rate between the moxibustion and western medicine groups (MD = 4.56, 95% CI [2.24, 9.26], I^2^ = 0%, *P* < .001; Fig. 8).^[12,14,17,18]^

## 4. Discussion

CP is a prevalent disorder that affects a large number of males across the globe.^[1,2]^ This condition is primarily characterized by the inflammation of the prostate gland, leading to various uncomfortable symptoms. Individuals with CP often experience pelvic pain, frequent urination, and sexual dysfunction, which significantly impairs their quality of life.^[2,3]^ To alleviate the symptoms and manage CP, pharmacological drugs are frequently prescribed.^[7]^ However, their effectiveness is often limited due to several factors. Firstly, the efficacy of these medications in relieving CP symptoms can be inconsistent, with varying results among different individuals. Additionally, the emergence of antimicrobial resistance has become a significant concern, as it hinders the ability of antibiotics to effectively treat CP. This resistance not only renders some medications ineffective but also leads to the potential for recurrent infections. Moreover, the administration of pharmacological drugs for CP management can come with undesirable adverse events. These side effects can range from mild discomforts to more severe complications, further hampering the treatment process. Additionally, the high costs associated with these medications pose a significant financial burden on patients, limiting their accessibility to proper treatment. Given these limitations, the exploration of alternative therapies for the management of CP becomes of paramount importance. Researchers and medical professionals continually strive to uncover innovative treatment options that can provide more effective and enduring relief for individuals suffering from CP. These alternative therapies may involve non-pharmacological approaches, such as physical therapy, lifestyle modifications, and dietary changes. Additionally, complementary and alternative medicine practices, including acupuncture, herbal remedies, and relaxation techniques, are also being investigated for their potential efficacy in relieving CP symptoms.^[11,15]^ Ultimately, the goal is to discover alternative therapies that not only address the underlying inflammation but also mitigate the associated symptoms, improve patient outcomes, and enhance their overall well-being. Through continued research and exploration, the medical community aims to revolutionize the management of CP and provide patients with more comprehensive and successful treatment options.

Moxibustion is a traditional Chinese medicine technique that has been used for centuries in the treatment of various health conditions, including CP.^[15–17]^ It involves the burning of dried mugwort, which is applied either directly on or near the body surface, to stimulate specific acupoints. Despite its long history of use, the effectiveness of moxibustion for CP has remained a subject of debate and uncertainty. Although previous clinical trials have investigated its effects on CP, the findings have been inconsistent and inconclusive. This lack of solid scientific evidence has made it difficult for healthcare professionals to confidently recommend moxibustion as a treatment option for CP. In order to address this gap in knowledge, a systematic review and meta-analysis were conducted. This involved a comprehensive search and evaluation of all relevant published studies on the use of moxibustion for CP. The results of these studies were then combined to provide a more accurate and reliable assessment of the effectiveness of moxibustion for CP. By pooling the data from multiple studies, the researchers were able to analyze a larger sample size and obtain more statistically significant results. The findings of the systematic review and meta-analysis provided a more comprehensive understanding of the benefits and limitations of using moxibustion for CP. The analysis explored various outcome measures, such as pain reduction, improvement in urinary symptoms, and overall quality of life. It also assessed the safety and potential adverse effects associated with moxibustion treatment for CP. Overall, the systematic review and meta-analysis revealed that moxibustion may have a positive effect on relieving pain and improving urinary symptoms in patients with CP. However, due to the limited number and quality of the included studies, more high-quality research is needed to confirm these findings and establish moxibustion as a reliable and effective treatment option for CP. In conclusion, although moxibustion shows promise as a potential treatment option for CP, the current scientific evidence is not yet strong enough to fully support its use. Further research and rigorous clinical trials are necessary to establish the true effectiveness and safety profile of moxibustion for CP.

This review conducted a comprehensive analysis of 8 RCTs involving a total of 664 participants. The aim of the review was to compare the effectiveness of moxibustion with other treatments commonly prescribed for CP, including herbal medicine and western medicine. After careful evaluation of the collected data, the analysis revealed significant differences in outcomes between moxibustion and the alternative treatments. Specifically, moxibustion was found to demonstrate notable improvements in the NIH-CPSI, which is a widely used tool to measure the severity of CP symptoms. This finding suggests that moxibustion holds promise as a potential therapeutic option for individuals suffering from CP. Furthermore, the review also discovered disparities in the overall response rates among patients who received moxibustion compared to those treated with herbal medicine or western medicine. This indicates that moxibustion may offer distinct advantages and beneficial outcomes for individuals dealing with CP. Overall, the findings of this analysis provide encouraging evidence supporting the use of moxibustion as a viable treatment option for CP. It highlights the potential of moxibustion to alleviate symptoms and improve the overall well-being of patients experiencing this condition.

It is important to acknowledge several limitations in this study. First, despite making efforts to search for relevant studies from reputable databases, it is crucial to acknowledge the possibility that some eligible trials might have been overlooked. It is possible that certain studies with valuable data or insights might have been unintentionally omitted, which could have influenced the overall findings and conclusions of this study. Second, it should be noted that the majority of the studies included in this research had larger sample sizes and stricter study designs. While this can contribute to the reliability and validity of the data, it is important to recognize that such study characteristics can also introduce biases in the overall analysis. For example, the outcomes observed in these studies might not accurately reflect the real-world scenario, as the strict criteria for participant selection and design might not be representative of the general population or everyday clinical practice. Lastly, the variations in the interventions and control modalities used in the included studies should also be taken into account. The differences in treatment approaches and comparison groups can potentially affect the results and introduce bias. The effectiveness of a specific intervention, for instance, might vary depending on the characteristics of the population or the context in which it is implemented. Therefore, it is essential to interpret the findings of this study with caution, considering the potential impact of these variations on the overall analysis and conclusions.

## 5. Conclusion

This study suggests that moxibustion may be a potential treatment option for male subjects with CP. However, due to the limitations mentioned above, cautious interpretation and further research are warranted to validate these findings and provide more robust evidence for the efficacy of moxibustion in the management of CP.

## Author contributions

**Conceptualization:** Xi-wen Yu, Cheng-si Wang, Xiao-hong Yu.

**Data curation:** Xi-wen Yu, Cheng-si Wang, Xiao-hong Yu.

**Formal analysis:** Cheng-si Wang, Xiao-hong Yu.

**Funding acquisition:** Xi-wen Yu.

**Investigation:** Xiao-hong Yu.

**Methodology:** Xi-wen Yu, Cheng-si Wang, Xiao-hong Yu.

**Project administration:** Xiao-hong Yu.

**Resources:** Xi-wen Yu, Cheng-si Wang.

**Software:** Xi-wen Yu, Cheng-si Wang, Xiao-hong Yu.

**Supervision:** Xiao-hong Yu.

**Validation:** Xi-wen Yu, Cheng-si Wang, Xiao-hong Yu.

**Visualization:** Xi-wen Yu, Cheng-si Wang, Xiao-hong Yu.

**Writing – original draft:** Xi-wen Yu, Cheng-si Wang, Xiao-hong Yu.

**Writing – review & editing:** Xi-wen Yu, Cheng-si Wang, Xiao-hong Yu.
